# Effect of two different bonding agents on the microleakage used for fluoride releasing pit and fissure sealant

**DOI:** 10.6026/973206300200855

**Published:** 2024-08-31

**Authors:** Prashant Jalannavar, Ketaki Rajguru, Sindhoori Goud, Soumyaranjan Nanda, Shreeyam Mohapatra, Ajit Kumar Swain, Pratik Surana

**Affiliations:** 1 Department of Pedodontics and Preventive Dentistry, PMNM Dental College and Hospital, Bagalkot, Karnataka, India; 2 Department of Conservative Dentistry and Endodontics, Tatyasaheb Kore Dental College and Research Centre, Nave Pargaon, Maharashtra, India; 3 Department of Conservative Dentistry and Endodontics, Mallareddy Institute of Dental Science, Hyderabad, Telangana, India; 4 Department of Conservative and Endodontics, SCB Dental College and Hospital, Cuttack, Odisha, India; 5 Department of Oral Medicine and Radiology, SCB Dental College & Hospital, Cuttack, Odisha, India; 6 Department of Conservative Dentistry and Endodontics, Kalinga Institute of Dental Sciences, KIIT deemed to be university, Bhubaneswar, Odisha, India; 7 Department of Pedodontics and Preventive Dentistry, Maitri College of Dentistry and Research Centre, Durg, Chhattisgarh, India

**Keywords:** Pit and Fissure Sealants, Bonding Agent, Microleakage

## Abstract

The evaluation of 5th and 7th generation bonding agents on the extent of micro leakage from sealant such as fluoride in forty extracted human premolar teeth is of interest to dentists. 40 extracted teeth satisfying the inclusion and exclusion criteria were randomly divided into two groups. Group I utilized a bonding agent from the fifth generation, whereas Group II employed a bonding agent from the seventh generation, both treated with Helioseal F Plus sealant. Following a 24-hour period of cultivation at a temperature of 37°C and subjecting the sample to 100 cycles of temperature fluctuation varying in temperature from 5°C to 55°C, the samples were submerged in a 0.2% methylene blue dye mixture for duration of 24 hours and examined under a microscope. Result showed that fifth-generation agent mean microleakage was greater, but not statistically significant. The seventh-generation agent demonstrated minimal microleakage with simpler application.

## Background:

Preventive dentistry has long emphasized the significance of sealant such as pit and fissure in teenager and children due to their profound impact on reducing the incidence of occlusal caries [1]. As technology advances, the quest for optimal materials and techniques to enhance sealant efficacy continues. The choice of bonding agent is a crucial component that significantly affects the efficacy of pit and fissure sealants, which functions to promote adhesion and minimize microleakage - a key contributor to sealant failure and secondary caries [2]. Bonding agents have evolved significantly over the years, with various generations developed to improve adhesive properties and simplify application procedures. [3] One-bottle systems, another name for fifth-generation bonding agents, combine the adhesive and primer in one fluid., necessitating an etching step but simplifying the process compared to their predecessors. In contrast, seventh-generation bonding agents, or "all-in-one" systems, amalgamate the etch, prime, and adhesive components into a single application process, representing a leap toward procedural efficiency and reduced technique sensitivity. [4] The tiny transport of ions, fluids, chemicals, or germs between the restorative material and the tooth is known as microleakage, remains a primary concern for the longevity and effectiveness of pit and fissure sealants. Fluoride-releasing sealants, such as Helioseal F Plus, offer the added benefit of fluoride release, which assists in remineralization and additional caries prevention, further necessitating the evaluation of bonding agents used in conjunction with these materials [5]. Therefore, it is of interest to document the effect of two different bonding agents on the microleakage used for fluoride releasing pit and fissure sealant.

## Materials and Method:

In the study, forty decay-free, complete mandibular premolar teeth that had been removed for orthodontic reasons were analyzed. The specimens were cleaned extensively after extraction to remove any last traces of blood, saliva, any periodontal tissue, pellicle, and calculus and plaque. So this protocol included the use of an ultrasonic inserts for precise removal of extraneous debris. As a precautionary step, the specimens were subjected to scaling and root planning procedure treatment combination of water and pumice, which is applied using a prophy cup. Subsequently, the samples had been thoroughly cleaned with water and then dried out with great care. For to maintain hydration and prevent brittleness, these samples were preserved in a solution of saline at ambient temperature. Samples were then categorized into two distinct groups:

Group I: Helioseal F Plus sealants with a fifth-generation bonding agent.

Group II: Helioseal F Plus sealants with a seventh-generation bonding agent.

First Category had the enamel painstakingly etched with 37% ortho-phosphoric acid, which was applied into every vulnerable pit and fissure right up to the cuspal inclines. After the etching process, the teeth were dried for duration of 30 seconds. Subsequently, they were cleaned using an air-water spray for a period of 20 seconds. Finally, the teeth were dried using oil-free compressed air. An evaluation was performed to ensure the enamel displayed a frosty-white appearance, indicative of proper etching. Afterwards, a fifth-generation bonding agent (3M ESPE Adper Single Bond 2) had been put to pit and fissures according to the directions provided by the company and cured employing a LED light-curing device.

In Group II, the fissures were promptly treated with a seventh-generation bonding agent (Coltene One Coat 7) in accordance with the directions provided by the manufacturer this was preceded by the application of an LED light-curing apparatus for curing. The Helioseal F Plus Sealant (Ivoclar) was then utilized to the fissures in a methodical manner, while adhering to the meticulous instructions of each manufacturer. In order to achieve a perfect application without any a periodontal probe gently pushed through the occlusal surface, looking for air bubbles or empty areas. Following sealant application, occlusal surfaces were treated with a LED unit, following recommendations provided by the manufacturer.

After the sealant was applied, the curing process was carried out with the help of the manufacturer's specified manual. Subsequently, the specimens were submerged in distilled water at a continue temperature of 37°C for a time period of 24 hours. Completing the incubation phase, the samples were subjected to thermocycling, which involved 100 cycles alternating between temperatures of 5°C and 55°C. Each cycle lasted for 30 seconds, mimicking the thermal stressors experienced in oral settings. Before doing the microleakage assessment, the root apices were effectively sealed using resin to prevent any dyed material from entering this area. In order to safeguard tooth surfaces that are not relevant, a dual layer of nail varnish was administered to whole tooth surface, leaving only a 2 mm border around the sealant application area. After preparation, the specimens had been submerged in 0.2% methylene blue dye for twenty-four hours to determine penetration. A diamond disk of double-faced was used to slice each sample longitudinally buccolingually after dye immersion. Slicing was carefully examined under a stereomicroscope at 40x magnification to detect microleakage. This thorough examination ensured an accurate evaluation of the sealant's efficacy in preventing microleakage around the treated fissures. The evaluation process was carried out by a sole assessor using a 4-point scoring system, employing the Ovrebo and Raadal criteria for assessing dye infiltration [6]. The criteria for evaluating microleakage are outlined as follows (Table 1).

## Results:

The average microleakage scores were greater for the teeth that received treatment with the fifth-generation bonding agent in comparison to those treated with the seventh-generation bonding agent. However, this difference did not have statistical significance (Table 2).

## Discussion:

The increased susceptibility to caries of the occlusal surfaces of posterior teeth-especially the pits and fissures-has long been known. The complicated organization of the occlusal pits and fissures is mostly responsible for the higher susceptibility. This problem is most noticeable in teeth that are erupting and going through the process of maturity, where anatomical features hinder effective cleaning, and incomplete enamel maturation further exacerbates caries susceptibility [7- 8]. The dimensions and contours of pits and fissures on occlusal surfaces exhibit variability; however they often possess a slender and winding nature, rendering them very suitable for the accumulation of food particles and microorganisms. [9] The toothbrush bristle diameter is usually around 0.2 mm, which makes it challenging to effectively remove food particles and microorganisms through mechanical means. In 1967, Cueto and Buonocore carried out the initial clinical experiment to assess the effectiveness of sealant retention. They found that one year after treatment, there was a significant 86.3% decrease in caries. Sealants are widely regarded as a highly effective supplement to preventive oral health care strategies for preventing tooth cavities [10]. In vitro tests can be used to assess microleakage in resin-based filling materials. The optimal resin-based filling material should have a minimum amount of microleakage. Ensuring accurate marginal adaptation is crucial for the durability of the sealant, as the infiltration of microorganisms beneath sealants triggers the formation of dental cavities [11]. The dye penetration method exhibits enhanced precision relative to alternative bacterial penetration techniques, attributable to the smaller diameter of dye particles compared to bacterial dimensions, as well as their comparable size to bacterial endotoxins. [12] In accordance with the scoring criteria established by Ovrebo and Raadal, this investigation conducted an in vitro evaluation of microleakage by evaluating the penetration of dye across the sealant and the tooth structure [6]. The present study evaluated the influence of fifth and seventh-generation bonding agents on the degree of microleakage in fluoride-releasing sealing agent applied to forty extracted human premolar teeth. Results showed higher mean microleakage for the fifth-generation agent, however, the observed distinction did not reach statistical significance. Fifth-generation bonding agents have various clinical drawbacks, including being time-consuming, expensive, and with dubious retention, so routine application of them in sealant procedures is not recommended. In contrast, utilizing seventh-generation bonding agents can significantly reduce both costs and time, as they eliminate the need for etching and rinsing steps. This decreases chair side time and increases patient comfort while also reducing contamination and increasing resin restoration effectiveness. Seventh-generation agents, sometimes referred to as self-etch adhesive bonding chemicals, may etch, disinfect, desensitize, prime, and adhere all in one process [13- 14].

## Conclusion:

The seventh generation of bonding agents exhibited negligible microleakage, showcasing a distinct benefit in procedural simplicity over their fifth-generation counterparts.

## Figures and Tables

**Table 1:** criteria for evaluating microleakage

**Score 0:**	**No dye infiltration.**
Score 1:	Dye infiltration confined to the outer half of the enamel-sealant interface.
Score 2:	Dye infiltration within the inner half of the enamel-sealant interface
Score 3:	Dye penetration extends into the underlying fissure.

**Table 2:** Descriptive Statistics

**Descriptive Statistics**	**Group I**	**Group II**
Number of Samples	20	20
Mean microleakage scores with Standard deviation	0.90 ± 0.45	0.35 ±0.40
Maximum	2	2
Minimum	0	0
P value		>0.05 (NS)
NS= Not Significant
